# MOGENS SCHOU: 100th anniversary of his birth

**DOI:** 10.1186/s40345-018-0138-4

**Published:** 2018-11-28

**Authors:** Paul Grof, Bruno Müller-Oerlinghausen

**Affiliations:** 1 Mood Disorders Center of Ottawa and Dept. Psychiatry, University of Toronto, Toronto, Canada; 2 Charité - Universitätsmedizin Berlin, Berlin, Germany


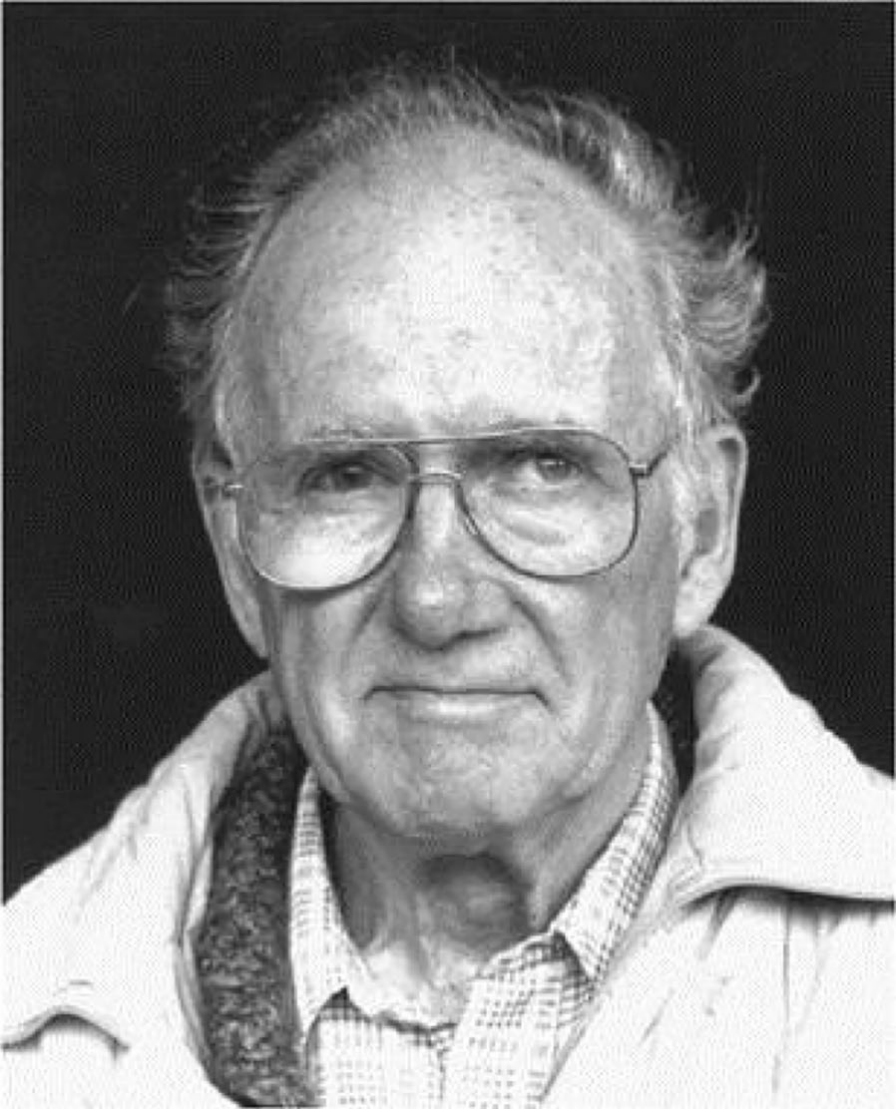


Mogens Schou (1918–2005)

On November 24, 2018, Mogens Schou would have turned 100 years old, and we wish to commemorate this special anniversary with thoughts about his personal and scientific legacy. To psychiatrists and neuroscientists his main contributions to the treatment of mood disorders are already well known and don’t need to be repeated. Notably recognized is Mogens Schou for discovering, in collaboration with Ch. P. Baastrup, the mood stabilizing effect of lithium treatment, for painstakingly investigating the various aspects of the practical use of lithium and for conducting the first double-blind clinical trial in psychiatry.

Much has already been written about Mogens Schou (Grof 2006, Schioldann 2006; Schioldann 2009) but if one wishes to learn about his life, it’s probably best to read what he has written about himself (Schou 2005). His autobiographical notes, written on the request of Johan Schioldann, document that his journey with lithium was an uphill push much of the time. We, two colleagues who collaborated with Mogens Schou very closely for several decades, may however offer a personal commentary about his legacy as they can be drawn from his life-long activities. We were tightly bound by the same interest—effective and safe management of recurrent mood disorders—and by friendship. When we ponder about his legacy for the near and outlying future of psychiatry and more generally of medicine, several points stand out in particular.

## Near future

The research tasks Mogens considered pressing are easy to name because he spelled them out himself in his last years. What he called his “Swan Song” had three main themes:

First, he was concerned that the evidence about the stabilizing effects of lithium in recurrent depressive disorders did not have much impact on clinical practice, despite many patients in need. He was distressed that the benefits of lithium in many recurrent depressions had not stretched to patients in need. Since then, however, this task is gradually being accomplished (Abou-Saleh et al. 2017).

Second, Mogens felt that the unique antisuicidal properties of lithium were still largely overlooked and, at his time, rarely quoted. While several researchers have mightily contributed to this discovery, by far the most extensive evidence came from IGSLI (http://www.igsli.org). Indeed, patients attempt or commit suicide much less often when they are on lithium than when they are not. Again, this effect of lithium has since Mogens’ passing been more widely accepted and referenced (Lewitzka et al. 2015). A common misunderstanding, however, is that this unique and specific effect not shared by any other compound refers exclusively to bipolar patients. But the truth is that also in unipolar patients lithium induces a dramatic reduction of the otherwise 2–3 fold elevated mortality (Abou-Saleh et al. 2017).

Third, he was also much preoccupied with a sufficient utilization of lithium in recurrent mood disorders. Fortunately, since that time there has been a palpable renaissance in the use of lithium. There are several reasons behind this development, in particular, a wider acceptance of its antisuicidal effects, the promise of neuroprotective benefits and its expanding value in neuroscience research. And recently the insufficient utilization, particularly in the United States, has been publicly acknowledged (Post 2018).

## International research teams

Schou developed various aspects of lithium’s acute and long-term treatment while leading a relatively restricted but very focused group of investigators. Parenthetically, it is striking that the major treatments that we have in psychiatric practice came from relatively limited, centered groups of investigators. However, Mogens also realized that there are many aspects of long-term treatment that can only be meaningfully researched in a well-integrated, multicenter, international collaboration. Therefore, in cooperation with the authors, in 1988 he founded IGSLI (International Group for the Study of Lithium treated Patients), and brought together seven centers having clinical research programs centered on lithium treatment and using the same diagnostic approach as well as a reliable documentation of their long-term patients treated mostly in specialized lithium clinics (Felber et al. 2018). For each research problem, one of the centers would lead and ensure the collection and data analysis in a uniform way. All members of the IGSLI consortium participated in the development of the design appropriate for the chosen hypotheses. Good examples of such IGSLI studies were, e.g. the investigations of the antisuicidal effects of lithium, its mortality reducing impact or the genetic studies of lithium-responsive patients.

None of the centers anywhere in the world has the number of research patients, capacity and the resources sufficient to investigate such problems on its own. Furthermore, no pharmaceutical company is interested in organizing or supporting such trials. Yet, together the centers were able to provide systematic, unified observations on a large volume of relatively homogeneous data. Many hundreds of lithium responders were followed for thousands of observation years. To share one’s own data with those of others in order to come to valid conclusions requires trust, if not friendship, among collaborators with no interfering conflicts of interest. Mogens always selected potential co-investigators in view of this basic requirement. The fact that the scientific findings published by IGSLI have never been opposed or falsified by other researchers underlines the importance of this principle. This IGSLI research concept may become a model from which other fields of medicine (e.g. the treatment of hypertension, diabetes or cancer) could learn.

## Bedside and bench

During the past half a century, much of psychiatric research was carried out separately at the bedside and in the laboratory. Mogens Schou was a prototype of a future researcher who was able to combine both; in the laboratory he led a team that focused on the basic science solutions to significant clinical problems he identified.

## Courage and persistence in innovation

Mogens was well aware that markedly advancing psychiatric research and significantly improving clinical practice requires innovative and courageous research. Yet, conceptual breakthroughs in science have always encountered intense opposition, refusal to change and sometimes even ridicule. Mogens faced plenty of these. From the initial observations of lithium’s stabilizing properties to its worldwide acceptance, it took him many years of a battle with resistance to his new concept of lithium stabilization. Future innovative researchers will encounter a similar opposition and will require an akin persistence.

Among other things, we need to be open to developing and experimenting with new methodologies for new problems. When Schou and Baastrup first observed the stabilizing effects of lithium, there was not a suitable strategy available for testing changes in the capricious course of recurrent mood disorders. Such strategies had to be formulated. The double-blind methodology was crucial for the final proof, yet Mogens Schou demonstrated that, when evaluating the changes in the recurrence of major mood disorders, comparable results were achieved in both double-blind and open trials in well documented patients. Such congruence, however, can only be expected if there is a real drug effect or clinically relevant effect size.

## Scientific communication

An important part of Schou’s legacy is clear and concise scientific communication, valuing the contribution of others carefully and highly. Exceedingly meticulous, Mogens was in the habit of rewriting many versions of each joint paper, until he considered the text crystal-clear. The scientific message must be unambiguous, the shortest possible, with words chosen economically.

A scientific lecture, according to Mogens must be dead on as to the allotted time. And this respect for the allotted time was strictly adopted by anyone who either had ever trained or had closely worked with him. He would have cut this Editorial to half-size.

## International recognition

For his discoveries and research Mogens Schou has received abundant recognition. He was given a large number of major awards including two nominations for the Nobel Prize. His research accomplishments have been recognized all over the world. He received several honorary doctorates and many awards and honors, including an honorary doctorate from Charles University in Prague, the oldest medical school in central Europe. He was proud and grateful for these accolades and appreciated the esteem of his colleagues. However, Mogens Schou vehemently protested the idea to honor him in the history of medicine by calling the lithium-responsive bipolar disorder Morbus Schou.

Finally, perhaps the most crucial part of his legacy were the personal qualities we all remember. They served him well in research, in collaborating and in communicating his finding. Dr. Samuel Gershon, an eminent researcher himself and the past president of the International Society for Bipolar Disorders, characterized well the loss we all encountered with Mogens: “Although his contribution to the field will leave a permanent and lasting legacy, the loss of his humanity and personal commitment to science and patient care are irreplaceable”.
